# Supplementary material for: The hot hand in the wild
Source: PLoS One. 2022 Jan 25;17(1):e0261890. doi: 10.1371/journal.pone.0261890 (PMC8789340; doi:10.1371/journal.pone.0261890)
Supplement: S1 Text — (PDF) [file pone.0261890.s001.pdf]

S1 Text. A roadmap/example for applying our statistical test. We will now describe in detail the process we follow to apply our statistical test on a specific player. Let us assume that we have a player, we call him Hooper, with data from 2 games. Each shot taken by this player had a shot make probability $p_{i,j}$, where i is the game the shot was taken and j is the sequence number of the shot within the game (e.g., $p_{2,3}$ is the make probability for the third shot of game 2). Table 1 shows the *input* data we will use for our statistical test for Hooper. For $k = 1$ we will use the shots that were taken after a make at the same game, which leaves us with the four data points with the asterisk in the table. As we can see two out of these four shots were made and hence, $\Pr[M|M]_{Hooper,data} = 0.5$.

Shot	Make Probability	Outcome
$p_{1,1}$	0.43	Make
$p_{1,2}$	0.31	Miss
$p_{1,3}$	0.17	Miss
$p_{1,4}$	0.96	Make
$p_{2,1}$	0.54	Make
$p_{2,2}$	0.37	Make
$p_{2,3}$	0.39	Make
$p_{2,4}$	0.42	Miss
$p_{2,5}$	0.48	Make

Table 1: Hooper's shots over a two-game span. The shots with the asterisk are the ones that will be used to assess the hot hand effect (for $k = 1$).

To calculate $\Pr[M|M]_{Hooper,sim}$ we sample the make probability for each one of these *qualified* shots. For example, for the second shot in game 1, we draw a random number between 0 and 1 uniformly distributed. If the number drawn is less than 0.31 (the make shot probability) we set the outcome for this simulation repetition 0. We repeat the process for the second, third and fourth Hooper's shot in game two, comparing the uniform random number drawn with the corresponding shot make probabilities (i.e., 0.37, 0.39 and 0.42). Let us assume that in this repetition the simulated outcomes for these shots are "Miss", "Make", "Make", "Make". Therefore, for this first simulation $\Pr[M|M]_{Hooper,sim} = 0.75$, which corresponds to an un-adjusted effect size of $0.75 - 0.5 = 0.25$. In order to adjust for the model error, we sample and simulate based on their shot make probability 4 randomly selected Hooper's shots (excluding the ones after a make used for the hot hand analysis) and calculate the difference between the simulated FG% and the one observed in these shots. For example, we randomly select the third and fourth shot from game one and the fist and fifth shot from the second game. Simulating them gives a simulated FG% of 75%, while the observed FG% in these shots is 75%; i.e., the model neither underestimated nor overestimated the FG% over these 4 shots. Hence, the adjusted hot hand effect size for this simulation is $\hat{e}_{Hooper,sim1} = 0.25 - 0 = 0.25$. We repeat this process 500 times and we calculate the average adjusted effect

size $\hat{e}_{Hooper,sim}$, while the p-value is obtained through the a one-sided t-test with $H_0 : \hat{e}_{Hooper,sim} = 0,\ H_1 : \hat{e}_{Hooper,sim} > 0$. For this example, the average adjusted hot hand effect size is approximately 1.4% with a p-value of 0.19 (i.e., there are no statistical evidence for Hooper exhibiting of hot hand).
