# Supplementary material for: The hot hand in the wild
Source: PLoS One. 2022 Jan 25;17(1):e0261890. doi: 10.1371/journal.pone.0261890 (PMC8789340; doi:10.1371/journal.pone.0261890)
Supplement: S2 Text — (PDF) [file pone.0261890.s002.pdf]

S2 Text. Model error adjustments. When adjusting the hot hand effect size for the error associated with the output of the shot make probability model, we sample a random set of shots for the player of interest to compare the actual FG% and the expected one over this set of shots. However, if any of these shots follows a sequence of made shots, then part of this error can be due to the presence of the hot hand effect, consequently leading to underestimating the effect size of the phenomenon.

To investigate this further we delved into the details of the prediction error for the shot make probability model. We define the model's error as the difference between the observed field goal percentage and the expected one. Furthermore we compute the model's error for two different classes of shots: **(A)** shots taken after a made one (this is the set of shots used for the hot hand analysis), and, **(B)** shots that do not follow a made one. In both cases, the model tends to underestimate the shot make probability, with an average value of 2.2% for case A and 1.6% for case B. The (paired) average difference is 0.6%, while the pooled average of these errors is approximately 1.9%. Consequently, n average the underestimation of the hot hand effect will be approximately 1.9-1.7 = 0.2%.

Based on the above, the results presented in this paper are conservative, in the sense that we would need to have stronger evidence to reject our null research hypothesis of no hot hand. Nevertheless, here we replicate the analysis by estimating the model error adjustment by only using shots that are not preceded by a made attempt (i.e., we eliminate the shots we use for the estimation of the hot hand effect). Our results are consistent with the aforementioned discussion. In particular, as we see from Table 1, there are now a few more players in each setting that have been identified as exhibiting the hot hand phenomenon. Again when we consider the whole league, players tend to have lower FG% after a sequence of makes than what is expected from the model.

k	# HH players	adj HH effect size	mean sequence length	overall adj effect size
1	41	0.023	480.5	-0.015
2	34	0.026	199.8	-0.027
3	33	0.041	81.9	-0.038
4	37	0.072	32.6	-0.047

Table 1: Adjusting for the shot make probability model errors using only shots not followed by a make provides us with similar results, and the same conclusions.
