# Supplementary material for: The hot hand in the wild
Source: PLoS One. 2022 Jan 25;17(1):e0261890. doi: 10.1371/journal.pone.0261890 (PMC8789340; doi:10.1371/journal.pone.0261890)
Supplement: Attachment — Submitted filename: PLOS_ONE_April_2021.pdf [file pone.0261890.s003.pdf]

Referee report for "The Hot Hand in the Wild"

April 2021

The paper tests the "hot hand" effect using a dataset from NBA shots but, unlike previous studies, relaxes the assumption that a player's shots are identically distributed. Instead, the paper uses information about each shot, such as distance to the basket, distance to the closest defender, touch time prior to shooting, or shot type, to construct a probability distribution about the chances that the current shot will be successful (a "make"). The authors then compare this probability against the observed probability, showing that the hot hand effect exists.

Main comments

1. The paper considers only two NBA seasons. Are they robust to data from more seasons, or are the results stronger in some seasons but weaker in other seasons? Given the applied nature of this paper, it could help the authors show that the results remain across multiple seasons.

2. From my interpretation, the authors calculate the probability distribution of a given shot being successful using the list of information in the bullet points of page 3 for each shot. What is a bit unclear is whether the probability of each shot being successful is drawn from this probability distribution (breaking the identically distributed assumption from other papers), whether they just use this probability to compare it against the observed probability that the shot was successful, or whether the authors do both. Please highlight which of these points the paper makes, as that will help differentiate it from the literature and, importantly, it will also help you convey the results of the paper to researchers in other fields.

3. It would be interesting if the authors can test their model in a free throw dataset. For a given player, his shots may not be identically distributed (with the probability of "make" in each shot being drawn from a different distribution, or alternatively, the conditional probability of "make" shot k is a function of the number of previous "makes" in the free throw contest.

4. In the last paragraphs of page 5, you mentioned that you also condition the probability of a make on whether the player made the last two shots as well, showing that your

results are qualitatively unchanged (although the intensity of the hot hand effect is diminished). It would be interesting, as a robustness check, if you can condition this probability on whether the player made a longer history of previous shots as well. Besides being a standard robustness check, it could help the reader understand how persistent the hot hand effect is, once a player starts experiencing it.

5. In the Discussion section, you mention several questions in the second paragraph, without offering some answers, or educated guesses, based on your results. From your findings, one could interpret that altering some of the independent variables listed in the bullet points of page 3 will affect the cumulative distribution function from where a player draws his probability of success in his next shot, ultimately affecting the emergence of the hot hand effect. If this interpretation is correct, then the authors should provide a clearer description of which variables listed in the bullet points of page 3 have the largest effect at the cumulative distribution function they construct. A clear understanding of these effect would provide more concrete policy implications, namely, which variables should a player (or a coach) affect to increase the chances of makes in each minute of a game.

Minor comments

1. Please define "relu" on page 3.

2. Please rewrite the paragraph at the bottom of page 3, as it's rather unclear.

3. Vector P is described in page 4 as "the vector with the shot make probabilities for each trial in the sequence". There is other, quite long, description of vector P in other sections of the paper. To simplify the notation, and clarify the intuition behind this vector, the authors could use notation from repeated games, calling P the "shot history."

4. The sentences starting at "Furthermore, from a technical standpoint, an ordinary least squares regression..." until "...estimation of the corresponding p-values" in page 7 are quite unclear. Please rewrite them to improve clarity. Are you refereeing to other papers in the literature using ordinary least squares in their regressions (that's what I think you mean) or did you use ordinary least squares at some point of your analysis?
