# Supplementary material for: The hot hand in the wild
Source: PLoS One. 2022 Jan 25;17(1):e0261890. doi: 10.1371/journal.pone.0261890 (PMC8789340; doi:10.1371/journal.pone.0261890)
Supplement: Attachment — Submitted filename: PLOSONE-HotHand.pdf [file pone.0261890.s004.pdf]

We would like to thank the reviewers for the useful comments. These comments have helped us to significantly improve - in our opinion - the quality of the manuscript. The additional results obtained as a consequence of the required changes have allowed us to refine our conclusions. The major changes we have made - among several changes detailed in our answers below and marked with red fonts in the revised manuscript - can be summarized in the following:

- We extended our analysis with data from the 2013-14 season. Thus, we now have 2 seasons of shots to run our analysis. In particular, in order to obtain the shot make probability for the shots during each season we train the model on the data from the other season. This ensures that the probabilities used are estimated out-of-sample. Then for each player we use their shot data from both seasons.
- We have added an adjustment at the estimation of the hot hand effect size to account for the errors introduced from the shot make probability model. In particular, the shot make probability is associated with some uncertainty/error for which we adjust.

Following are our detailed responses to each reviewer's comments/questions.

<u>Reviewer 1</u>

1.The paper considers only two NBA seasons. Are they robust to data from more seasons, or are the results stronger in some seasons but weaker in other seasons? Given the applied nature of this paper, it could help the authors show that the results remain across multiple seasons.

We would like to thank the reviewer for this comment and we agree that an analysis including more seasons would add robustness in the observed results. However, unfortunately we only have 2 years of tracking data for the shots. While it is possible to obtain shot locations and outcomes for multiple years from play-by-play data, these do not include crucial variables like closest defender distance, touch time before the shot, dribbles prior to the shot, defender ID etc. Therefore the quality of the shot - i.e., the make probability for a shot - cannot be assessed accurately. Nevertheless, in the first version of the manuscript we trained the shot make probability model on the data from the 2013-14 season and then run the "hot hand" analysis on the 2014-15 season in order for the predictions from the model to be out-of-sample. In the revised version of the manuscript, we trained again the shot make probability model using the same neural network architecture on the data from the 2014-15 season and ran the "hot hand" analysis on the 2013-14 season as well. This way the predictions from the model are still out-of-sample, while we have now a larger observation set from players from 2 seasons.

2.From my interpretation, the authors calculate the probability distribution of a given shot being successful using the list of information in the bullet points of page 3 for each shot. What is a bit unclear is whether the probability of each shot being successful is drawn from this probability distribution (breaking the identically distributed assumption from other papers), whether they just use this probability to compare it against the

observed probability that the shot was successful, or whether the authors do both. Please highlight which of these points the paper makes, as that will help differentiate it from the literature and, importantly, it will also help you convey the results of the paper to researchers in other fields.

We would like to thank the reviewer for bringing up this issue, which allows us to clarify. We have better explained this part in the revised manuscript, but in brief, this model outputs a “make” probability for each shot in our dataset. We use this probability to simulate this same shot several times - i.e., through a “biased coin” according to the shot make probability - and compare the simulation results from a series of shots, with the actual outcome of the same series in our dataset.
One possible point of confusion might be the use of the term “observed” probability for the calibration curve of the model. This is purely for evaluation purposes of the shot make probability model, and it is the standard approach and terminology for probabilistic model evaluations. In particular, we cannot know the **true** probability of a shot being made. However, we can group shots with the same/similar *predicted* probability and then obtain the observed probability as the percentage of these shots actually made. For example, if we take all the shots where the model predicts a make probability of 55%, then the observed probability is simply the percentage of these shots that were actually made during the games. We have clarified this point further in the revised manuscript.

3.It would be interesting if the authors can test their model in a free throw data set. For a given player, his shots may not be identically distributed (with the probability of "make" in each shot being drawn from a different distribution, or alternatively, the conditional probability of "make" shot k is a function of the number of previous "makes" in the free throw contest.

We would like to thank the reviewer for this comment. This analysis is certainly possible, and FTs are one of the settings examined in the hot hand literature. While the tracking dataset we used in our manuscript does not include free throws, obtaining FTs is possible through play-by-play data. The challenge with FTs is that they are with high confidence identically distributed. Several different splits (e.g., home-away, first-second half etc.) provide a similar overall FT% for a player, allowing us to treat all FTs as identical trials. Thus, Miller’s and Sanjurjo permutation test is directly applicable here. We have run the permutation test on FT data from the 2019-20 season by randomly permuting FTs within the same game and using the sequence from all the games to estimate the probability Pr[Make FT_i | Make FT_{i-1}]. For example, LeBron James has a 71.8% probability of making a free throw after he made his previous one, which is higher compared to the 66.9% that we would expect at random - i.e., after permuting his FTs - with a p-value of 0.003. One the other hand, Steven Adams, does not exhibit the hot hand at FTs. He makes 52.3% of his FTs after a make, which is statistically indistinguishable (p-value 0.26) from the 49.2% expected from random permutations of his FTs. The results from the FTs are similar with the results from in-game shots, in the sense that there are players with strong effect and others with no effect. However, given that this analysis does not require removal of the assumption of identical trials, we decided to not include these results

in the revised manuscript as we felt that it might break the flow of the study and confuse the reader in terms of the objective of the study, which is tied to the non-identical nature of the trials.

4.In the last paragraphs of page 5, you mentioned that you also condition the probability of a make on whether the player made the last two shots as well, showing that your results are qualitatively unchanged (although the intensity of the hot hand effect is diminished). It would be interesting, as a robustness check, if you can condition this probability on whether the player made a longer history of previous shots as well. Besides being a standard robustness check, it could help the reader understand how persistent the hot hand effect is, once a player starts experiencing it.

This is a very interesting point and indeed being able to condition on long series of successes is going to provide additional insights on the persistence of the phenomenon. In the original manuscript, given that we used one season of data to analyze/quantify the hot hand phenomenon the sample sizes became very small for larger values of k. However, in the revised manuscript we have used two seasons of data and by reducing the constraints for players qualified for our analysis (in terms of total number of shots) we were able to examine values of consecutive makes up to 4. The results show that the phenomenon is persistent for individual players. We have added a detailed description of these results in the revised manuscript.

5.In the Discussion section, you mention several questions in the second paragraph, without offering some answers, or educated guesses, based on your results. From your findings, one could interpret that altering some of the independent variables listed in the bullet points of page 3 will affect the cumulative distribution function from where a player draws his probability of success in his next shot, ultimately affecting the emergence of the hot hand effect. If this interpretation is correct, then the authors should provide a clearer description of which variables listed in the bullet points of page 3 have the largest effect at the cumulative distribution function they construct. A clear understanding of these effect would provide more concrete policy implications, namely, which variables should a player (or a coach) affect to increase the chances of makes in each minute of a game.

We would like to thank the reviewer for this comment. Our objective with posing these questions in the discussion is to point towards some directions for future research. Unfortunately, we are not really able to identify the underlying mechanisms (physiological, neurological or psychological) that lead to the emergence of the phenomenon for some athletes with the observational data of makes and misses. As we mention in the revised discussion the emergence of the hot hand phenomenon in game-situations might actually not even have to do anything with factors that come to our mind when we talk about the hot hand, such as psychological, but rather be a result of a player being able to *hunt* and exploit favorable matchups consistently during a game. While we can not provide answers to the questions described at this part of the manuscript, we believe it is useful to pose these questions in order

to lay a set of potential paths for other researchers (as well as our own future work) to take on. We have re-edited that part of the manuscript to clarify our objective with these questions.

Furthermore, the shot make probability model is certainly crucial for the results from our analysis. An inaccurate model will lead to over- or underestimation of the phenomenon, depending on whether the model under -or over-predicts the shot make probability respectively. In the latest manuscript we have revised our framework to include an adjustment for possible errors associated with the shot make probability model.
An interesting, tangential to the reviewer's point here is the *reaction* of the players themselves to consecutive makes. As Bocskocsky et al. (2014) showed, when a player makes a shot they tend to take tougher shots next, i.e., shots with lower probability of success (e.g., shots further away from the basket, heavily contested etc.). However, our analysis adjusts for this, since the simulated makes/misses are drawn from the shot make probability model.

Minor comments

1.Please define "relu" on page 3.

We have defined the relu function.

2.Please rewrite the paragraph at the bottom of page 3, as it's rather unclear.

We have edited the paragraph, and we believe it is more clear now.

3.Vector P is described in page 4 as "the vector with the shot make probabilities for each trial in the sequence". There is other, quite long, description of vector P in other sections of the paper. To simplify the notation, and clarify the intuition behind this vector, the authors could use notation from repeated games, calling P the "shot history."

We would like to thank the reviewer for this suggestion, which we have followed in the revised manuscript.

4.The sentences starting at "Furthermore, from a technical standpoint, an ordinary least squares regression…" until "…estimation of the corresponding p-values" in page 7 are quite unclear. Please rewrite them to improve clarity. Are you refereeing to other papers in the literature using ordinary least squares in their regressions (that's what I think you mean) or did you use ordinary least squares at some point of your analysis?

We would like to thank the reviewer for this comment. We indeed mean that the prior literature cited has used OLS. We have updated this part of the manuscript to make it more clear.

<u>Reviewer 2</u>

1. The authors' analysis is limited to two seasons of NBA data, which results in shot sample sizes for individual players that the authors state are too small to test for shooting performance on streak lengths longer than two. They offer no explanation for why they did not consider more years of data. Using more data would be nice for a number of reasons. For one, the tests would be more powered on the individual level. Second, they could consider performance on streaks of length three, which is fairly standard in the literature. Third, they could consider more individual shooters than the 21 that they currently consider (they should explain how they selected the 1000 shot inclusion criterion, and the sensitivity of their results to it). This would make their tests more robust, and the selection of shooters would be more representative of the typical shooter in the league. I hope that the authors can obtain more data and extend their analysis to the larger set of data.

We would like to thank the reviewer for this comment. We agree that having more data would help in several fronts as detailed in the comment. However, tracking data are publicly available only for the two seasons we have access to. The NBA has made this detailed information about shots private/proprietary after the 2014-15 season. Therefore, adding more data in our analysis is not possible. However, we updated our analysis in order to increase the sample size - both for individual players and in terms of players examined - and to allow us to perform some of the things recommended by the reviewer's comment. In particular, in the original manuscript we only used the 2014-15 season for examining the hot hand. The thought process is that we will train (test and validate) the shot probability model with the 2013-14 season data and then evaluate the hot hand on an out-of-sample dataset. In the revised version of the manuscript we still evaluate the hot hand using out-of-sample data, but we trained two separate models using the same neural network architecture. The one is using the 2013-14 season's data to learn the model weights and then make predictions for the shot make probability of the 2014-15 season shots, while the second model is trained on the data from the 2014-15 season and used to estimate the out-of-sample shot make probability for the 2013-14 season. This way there is no data leakage from the training of the model to the predictions for the shot making probability in the player/shot sequences of interest.

Furthermore, we agree that our initial filtering process was too stringent. In the revised manuscript, we have loosened the threshold for the qualified players to be included in our analysis, by filtering out players with less than 1,000 shots over **both seasons** in the data. The reason for using this filter is that by allowing players that do not meet this criteria, we will end up including in our analysis players that took on average less than 6 shots per game. These sequences will not provide usable data, particularly for larger values of streak length. This filtering leads to 153 qualified players (as compared to the 21 players included in the first version of the manuscript), while it also allows us to examine streak lengths up to 4.

2. As mentioned above, the authors train their model of hit probability for each shot type for each player using one season (the first) then use this to build null distributions for testing shot performance of the players in the second season. I understand that this may be standard procedure for "hold-out" out-of-sample testing, but I don't understand why the authors do not perform robustness checks. For example, cross-validation makes sense to me here, starting with instead using the second year to train the model, then test on the first year of shooting performance. Also, is it overly problematic to train the model on both years of the data then test it on performance from both periods (at least for a robustness check)? In principle, this would seem to allow for a more apples-to-apples analysis given that the model would be better calibrated to the "true shot probabilities." Also, this would allow more shot data to be used in tests on performance. Though as mentioned in comment 1, I hope the authors can obtain and analyze more data.

We would like to thank the reviewer for this comment - which is relevant to the first one. Using in-sample predictions could lead to understating the hot hand effect since the predicted probability would be biased towards the actual outcome of the shot (either made or missed). Now, of course the question is how much will this bias be, and the answer here is not trivial. After the reviewer's comment we actually experimented with a leave-one-out setting (i.e., train a model with all the data, and one with all the data but one particular shot) and we found that the bias does not appear to be more than half percentage point in the majority of the cases examined[1]. However, in our case this could still be a large bias relative to the effect size of the hot hand (approximately two percentage points). Therefore, we decided to stay with out-of-sample predictions for the hot hand. Nevertheless, as detailed in our answer on the first comment, we have now used more data to train the model.

Moreover, while training the neural network we have used a validation set for early stopping. Finally, we performed some robustness checks by training the model on the 2013-14 (2014-15) season and evaluating on the 2014-15 (2013-14) season, and the performance in terms of accuracy, Brier score and validation curve is practically indistinguishable. In the revised manuscript, we have included the results from the updated process described in our response on the first comment.

Other Comments:

1. The authors quickly mention that if they were to have instead run a permutation test on shooting performance when on streaks of hits, permuting on the game x individual level, then they would have found much less evidence of streak shooting. This is a bit misleading in the sense that permuting on the game level eliminates the possibility of detecting any hot hand that initiates between (rather than within) games, whereas the authors' primary analysis does not. Thus, this is a bit of a stacked comparison. In Miller and Sanjurjo's working papers on controlled shooting experiments (R&R at Review of

---

[1] We have not included the results in the manuscript, since we thought that it would be rather confusing for the readers. However, we think that this can by itself be a research question: while we know that aggregate in-sample evaluations are biased, how much is the bias for a single data point?

Economics and Statistics) and the NBA Three Point Contest (forthcoming at European Economic Review) they perform robustness checks in which they consider more granular permutation strata, e.g. for contest year, shooting round, ball on rack within round, and so on. Stratifying on the "contest x round" level (which would be the most similar to permuting on the game level in the authors work, tends to reduce statistical significance, for the reasons they discuss: it desensitizes tests to both: hot hand that activates between rounds, as well as other systematic changes in shooting behavior that activates between rounds not due to the hot hand. In this sense it is conservative to permute on the more granular levels. The authors should qualify their discussion accordingly.

We would like to thank the reviewer for this thoughtful comment. Indeed, our analysis is only able to identify hot-hand effects within a game since we are not considering consecutive shots across games. While in a three-point contest examining streaks that span consecutive rounds (rather than only shots during the same round) makes a lot of sense -- similar to a game situation and halftime for example -- in game performance it does not seem to be particularly relevant. However, we agree that this approach would provide conservative estimates if there is hot-hand that activates between rounds/games. Nevertheless, when estimating the effect for individual players, we use the whole sequence of qualifying shots (i.e., a shot taken after k makes) with the constraint that all these k+1 shots need to be within the same game. This provides us with fairly large sequences/sample size (particularly for k = 1 and k = 2) that allows us to avoid statistical power issues for smaller sample sizes of binary data.
Furthermore, we would like to clarify that in our uniform permutation test (i.e., without considering the shot quality), our goal was not to per se compare with Miller and Sanjurjo's work directly in terms of the conclusions about the presence of hot hand or not, but rather to showcase that ignoring the shot quality can lead to underestimation of the effect. Again during this permutation we consider all qualified shots for a given player, but we permute only shots within the same game. We have added and clarified these points in the revised manuscript as per the recommendations of the reviewer.

2. It seems it would be worth adding a bit of discussion on the variables the authors use vs. those used by Bockocksky et al, and Rao before them, and explaining why there are differences, if there are.

We have added some discussion on the similarities and differences between our shot make probability model and those from A. Bocskocsky et al., as well as, Daly-Grafstein and Bornn. In brief, compared to the model from Bocskocsky et al., there is quite a bit of overlap in terms of the variables used, but there is a clear difference in the modeling approach. On the other hand Daly-Grafstein and Bornn, make use of more granular tracking data, and in particular the actual trajectory of the ball after a shot, from which they extract variables such as the entry angle to the hoop, the distance from the center of the hoop etc. These variables are not available at the (public) dataset that we used.

3. There are a few things the authors can clean up: (i) the discussion of base rate vs. p(M|M)_perm vs. p(M|M)_data can be written better; as is it is a bit confusing, (ii) The discussion on the stability of effect size for streaks of length one vs two is potentially a bit misleading; depending on the model of hot hand shooting, the extent of hot hand expected on each of these two shot situations could easily be different, and there is an attenuation bias in true effect size due to measurement error (which varies with streak length) that is pointed out in the literature in work by Arkes, Stone and Arkes, and in each of the papers of Miller and Sanjurjo, (iii) the second para of the intro is written as if GVT were unaware of potential confounds in game data; this is misleading in the sense that they acknowledge this so consider also free throw shooting and conduct a controlled shooting experiment, (iv) similarly, say "free throw attempts or three-point contests are typically used when studying the phenomenon in basketball"; here, controlled shooting studies are excluded; the authors should consider citing the papers by koehler and Conley and Miller and Sanjurjo on 3pt shooting contest, and the controlled shooting study of Gilovich et al, and the analysis of several controlled shooting studies in another paper by Miller and Sanjurjo. Similarly, this may be the place to quickly cite other work on NBA game shooting, (v) (last para of the Intro) the authors state that permutation tests are common in hot hand studies with basketball data (and in discussion say "permutation tests have been used by the majority of the hot hand literature.."). They are used in Miller and Sanjurjo's work, but were not used in GVT and those that followed, until the recent work; in particular, who has used permutation tests on game data, as the authors suggest?, (vi) in the same paragraph the authors suggest that permutation tests are vulnerable to the small sample bias observed in Miller and Sanjurjo (2018), but those authors make clear that permutation tests under the i.i.d. assumption are not vulnerable to the bias. The authors should make clear what biases they are referring to. As written "b" does not seem correct to me. As written it seems possible that GVT conducted permutation tests that were vulnerable to the small sample bias. This is not the case, (vii) the small sample bias does not just appear in small samples; it appears in all samples but is more pronounced in small samples.

We would like to thank the reviewer for this detailed comment on a few misrepresentations we had in the first version of the manuscript. We have updated the revised manuscript accordingly and in particular:

(i) We have updated the discussion on our simulations/permutations
(ii) This is a particularly important issue that we thought more about after the reviewer's comment and led to one of the major changes in the revised manuscript. In particular, and as detailed in our responses above, we have added a mechanism for adjusting for the shot make probability model's errors with a detailed description in the revised manuscript.
(iii) This was certainly not what we wanted to imply. However, we see now how this could be perceived this way. We have re-written that part of the introduction to clarify.
(iv) We have included studies on controlled shooting, which we missed in our initial version
(v) We erroneously used the term "permutation tests" in this part of the paper. We were referring in general to analysis of binary sequences that can include permutation tests, runs tests etc. We

have changed the wording at this part to clarify that we are talking about combinatorics of binary sequences in general.
(vi) We have re-written this part to clarify our point. Our point was not that the permutations tests are vulnerable to the small sample bias observed by MS. We were referring to biases from assumptions that each trial is identical (i.e., same probability of success). We have updated the text accordingly
(vii) We have also clarified this point.

4. The writing can be polished a bit. For example, "extent" rather than "extend", "shot" not "show", "sampling this process...several times" should be more explicit, e.g. 10,000 times, I´m not sure "robust" is the right word when talking about whether the hot hand is common across players, "in the different.", "is less than 1&", "decisions making"

We have made a careful editing of the revised manuscript, fixing a lot of the typos and grammatical errors in the first version.
