# Supplementary material for: The hot hand in the wild
Source: PLoS One. 2022 Jan 25;17(1):e0261890. doi: 10.1371/journal.pone.0261890 (PMC8789340; doi:10.1371/journal.pone.0261890)
Supplement: Attachment — Submitted filename: HotHand-PLOSONE-R2.pdf [file pone.0261890.s005.pdf]

We would like to thank the reviewer for their additional comments that have helped us further improve the manuscript. While we provide a point-by-point response in each comment/question from the reviewer, here we summarize the major changes we performed during this revision:

- We have added supplementary material for describing the detailed process of performing our statistical test
- We have added supplementary material for exploring other approaches to adjusting for the shot make probability model error
- We have also expressed our conclusions more clearly and tied them with existing literature (and in particular the Lantis and Nesson paper, which somehow we had missed). As part of this point we have also made sure that we do not overstate/overgeneralize any of our conclusions/results.

In what follows we provide a detailed response to the comments and describe the changes we have performed in the revised manuscript.

1. The authors need to do a much better job of relating their work to the previous literature. For example, their work is quite closely related to that of Rao (2009) and Lantis and Nesson (2019). Neither of these papers is cited.

Lantis and Nesson (2019) study 12 years of NBA shooting data, including the 2 years studied by the current authors. In addition, they study both field goal shooting and free throws, as opposed to just field goal shooting. Further, they use a considerably longer list of controls than the current authors.
Their pooled test results are similar, and they don't have tests on the individual level like the current authors now do.

The authors cite Bocskocsky et al. (2014) as if it is perhaps the only regression analysis that has been performed in the hot hand fallacy literature. This is very misleading. Arkes, Lantis and Nesson, Green and Zweibel, Miller and Sanjurjo, and others use regression analysis as well.

The authors should make this clear. It is a valid, and typical, approach used in the literature. Multiple approaches have been taken to correct for the bias pointed out by Miller and Sanjurjo. The particular weaknesses of Bocskocksky et al. are not general to these studies.

An obvious question is what result would these other typical regression approaches produce with the same data. Lantis and Nesson provides an answer to this question, with more data.

We would like to thank the reviewer for pointing out these omissions in our literature review. While we were aware of Rao (2019) we felt it was more focused on the players' perception of

the existence of the hot hand - as captured from their shooting choices/behavior following a make or miss - while it was based on data from 8 players over 60 games. On the contrary, we had missed the Lantis and Nesson (2019) paper. This is a very interesting paper that overall is in agreement with our findings. In particular, they mention that when using the data from the two seasons we also use, and hence, they can control for the shot quality, overall there is a negative effect from a streak of successful shots. This is in alignment with our “league-wide” results. Furthermore, referring to Simpson’s paradox, they mention that this does not preclude individual players from exhibiting the hot hand phenomenon - something that we examined and indeed is the case for specific players. We have added a discussion for these papers in the revised manuscript.
Furthermore, indeed we were referring to the weaknesses of the regression approach with regards to the setting at Bocskocksky et al. and not in general in the use of regression analysis in the study of the hot hand. We have clarified this in the revised manuscript.

2. If I understood the authors' empirical approach correctly, they are comparing the computed P(M|M...M)_sim in (heterogenous Bernoulli) simulated datasets to the observed P(M|M...M)_data in the real data, rather than comparing P(M|M...M)_data to performance on the directly matched shots taken on the same streaks the real shooter was actually on. The latter approach seems more direct, and that it will be less suceptible to measurement error. The former approach may even be suceptible to systematic bias if the types of situations in which real shooters go on streaks create systematic differences in the sampling of their streak shots and those in the simulated datasets.

I suggest that the authors run the analysis I mention, and compare results with those of their current analysis, or if they agree that it is better, replace the previous analysis with it. (Later, I see new added text "We then simulate his shot sequence after k consecutive makes 100 times..."; this suggests the latter approach; this should all be explained much clearer, so there is no ambiguity)

We would like to thank the reviewer for this comment and apologize for any confusion. We are indeed comparing the simulated heterogenous Bernoulli with directly matched shots taken by the shooter in games. We have updated the corresponding text and we believe it is much more clear in the revised manuscript.

3. Relatedly, in the revision the authors apply an ad hoc model error adjustment that samples from all shots (not just streak shot situations), then apply it to adjust hot hand estimates. First, this is a potentially serious issue with the modeling approach that the authors should address in a more complete way. Why is this happening? Is it happening systematically across shooters? If so, why? Second, if this adjustment samples at random from all shots (including shots taken on streaks) then this is creating in turn another mechanical bias against hot hand effect size. An alternative would be to sample without including these shots, which is not ideal for other reasons, but perhaps better. In either case, because this adjustment is non-trivial in size, if the authors are going to use it, they should make sure that it is not biasing hot hand effect sizes in some other more

subtle way. For example, they can run simulations using different models of hot hand shooting (that is, in which they have aissumed there are hot hand shooters), and see if their model systematically produces errors in the same direction. If so, this would suggest that the measurement error they are picking up is actually a mechanical artifact of hot hand shooting, so making the correction they are could bias results against finding the hot hand.

Are the errors similar in both directions when the authors train the model in one direction, then the other?

If we are understanding this comment correctly, the main point the reviewer brings up is the fact that the model error includes any hot hand effect if present, and by using a random sample of shots to estimate the expected error for the model we could bias this estimation since it will/can include shots that have been affected by the “hot hand” (if the phenomenon exists). This essentially can lead to an underestimation of the hot hand effect. We agree with this and it is a very crucial point that we address in what follows.

In general, the typical sources of model error are present in our shot make probability model as well, including things like omitted variables, measurement errors (e.g., in this case, shot locations might not be accurate for data obtained from specific arenas, or logging of shot types might introduce errors/inconsistencies etc.) etc. These sources of error are not systematically present in specific types of shots, but all shots in our data should be affected (or better put it is realistic we expect to be affected) in the same way. However, on top of these error sources, shots taken after a sequence of made shots might exhibit an additional error source, namely, the effect of the hot hand phenomenon (if true). Consequently, if these shots are used to estimate the expected model error - under the assumption that consecutive makes do not correlate with the success of the following shot - they can bias this estimate if the hot hand exists.

To investigate this further we examined the model errors in more detail. We define the model error as the difference between the observed field goal percentage for a player over all his shots and his expected field goal percentage. For a given player in the set of players examined, we estimated two different types of model errors: (a) error e1 for shots following successful shots (this is the set of shots used for the hot hand analysis), and, (b) error e2 for those that do not. In both cases, the model tends to underestimate the shot make probability, with an average value for e1 = 2.2% and for e2 = 1.6%. The (paired) average difference e1 - e2 is 0.6% (the following figure provides the obtained histogram). The pooled average of these errors is approximately 1.9%, which means that on average the underestimation of the hot hand effect will be approximately 1.9-1.7 = 0.2% (if we assume that all the error term for the shots we use to examine the hot hand effect is associated with the hot hand).

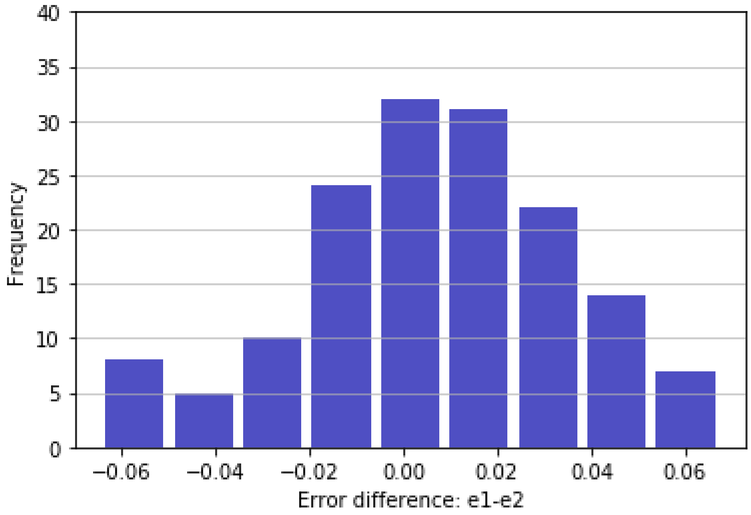

While the aforementioned shows that we might be underestimating the hot hand effect size - or even the presence of the phenomenon altogether - we are being conservative in our analysis, in the sense that we would need to have stronger evidence to reject our *null research hypothesis* of no hot hand. Therefore, while we keep this adjustment (i.e., using a random sample of all the shots) in the main text of the manuscript, we provide the same analysis in the supplementary material by only using shots that we do not use for the estimation of the hot hand effect. Our results agree with the aforementioned discussion, in the sense that there are now a few more players in each scenario (i.e., different value of k) exhibiting the hot hand phenomenon, but overall, when we consider the whole league, players tend to have lower FG% after a sequence of makes than what is expected from the model.

Finally, while our results/numbers mentioned above refer to the aggregate data from both seasons in the dataset, we have examined the same for each season individually and there are not any statistical differences.

4. in the KW example how is it possible that he only has 333 shots following at least one make, if he has taken more than 1000 shots? The only explanation that seems to make sense is that the authors are considering streaks of exactly one hit, rather than of at least one hit. But conditioning on at least one hit makes more sense because it provides more powered tests. The same goes for longer streak lengths.

We would like to apologize for any confusion. As we mention in the text, if for a specific shot there are missing features needed for the shot make probability model to make predictions or any shot for a player during a specific game is missing, then we eliminate from our dataset all the shots taken by this player in that particular game. For example, if Kemba Walker took 20 shots in a game and his shot #15 is either missing from the data or we do not have all the features to estimate a shot's probability, we remove all 20 shots from our analysis. So while we filter players with at least 1,000 shots over the two seasons that our dataset covers, the actual number of data points we can use is effectively smaller. Furthermore, assuming that there is not any systematic bias in the missing shots, players with more shots in general will also have more

shots missing from the database, removing several of their games from our final dataset. In particular, for Kemba Walker we end up with a total of 844 shots. Given that his overall field goal percentage during the two seasons covered was approximately 39%, 333 shots is a *consistent* value for the number of shots after a make. We have updated the description of our filtering process to make this more clear.

5. The authors should discuss how using one year to train a model to make probability estimates for another year can affect their results if there are systematic differences in shooting performance from one year to the next, e.g. the shooter improves, or changes technique over the offseason.

This is certainly one potential source of model errors (related to question 3 as well). However, we believe that in our case this does not pose a major concern for the following reasons:

1. The year-to-year correlation for the field goal percentage of players is fairly high. In our data, the correlation for the players with at least 1,000 shots across the two seasons (i.e., those we used for our analysis) is 0.76. Of course, the field goal percentage is a combination of both skill and shot selection. Nevertheless, this high correlation points towards large changes in the field goal percentage from year-to-year being mainly outliers.
2. While figure 1 includes the out-of-sample model evaluation for the pooled data from both seasons, we have also obtained the same evaluation results for each season individually and there is no statistical difference between the two. This is *consistent* with a setting where there are not significant changes in the shooting skill of player on average.

Of course, it is still possible that for specific players this setting might over or under-estimate their shot make probability. However, due to the reasons aforementioned, we do not anticipate any systematic differences on average. Anecdotally also it is extremely rare to see a player significantly improve his shooting over a single offseason (of course, a gradual improvement is highly possible -- if not expected). The only such examples that comes to our mind is (a) Lonzo Ball who shot 37.5% from the three point line during his first season with the Pelicans, after shooting 33% with the Lakers the season before, and, (b) DeAndre Jordan who made a leap in his free throw percentage during two consecutive offseasons (from 2016-17 to 2017-18 going from 48% to 58% and then in 2018-19 shooting free throws at 70.5%). We have a small discussion in the final section of the manuscript.

6. The sampling without replacement example to explain the bias discovered by Miller and Sanjurjo is not correct. In the Econometrica they show that the bias is generally larger than the analogous sampling without replacement type effect.

We would like to thank the reviewer for this comment, which allows us to clarify the inclusion of this example. Our goal with this example is not to provide a detailed *proof* of the results from Miller and Sanjurjo but rather to provide the high level intuition of the streak selection bias. Now having said that, indeed, given that our example considers cases of k=1, the streak selection

bias can be thought of as a form of sampling-without-replacement bias (Appendix D of the Econometrica paper), while for k > 1, there is an additional source contributing to the streak selection bias, namely, sequence overlap. The latter is absent in our example. However, as aforementioned our goal is to provide some basic intuition on the main result from Miller and Sanjurjo, which can be surprising for people unfamiliar with the area. We have clarified in the document that this example is just showcasing one source of the streak selection bias, while we provide some pointers at the Econometrica paper for more technical details for the interested reader.

7. In response to point 3 of the other reviewer, the authors argue that an analysis of free throw shooting is not interesting for them to include because--unlike field goals---free throws are essentially identically distributed. This is not correct. Players shoot systematically better on the second ft than the first, for example.

This is indeed the case; there is a quantifiable and robust difference for the free throw percentage of the first and second free throw (about 4 percentage points) during a free throw trip[1]. However, in our opinion, this process is still not as heterogeneous as shots taken during live ball action in a game. Most probably, if we were to order the settings with regards to *heterogeneity*, free throws are in-between controlled shooting experiments (more homogeneous) and live ball field goal attempts (more heterogeneous). They are an interesting setting, but we felt that adding these results could overload the paper and disrupt its flow. However, if the reviewer feels strongly about adding these results we can try and find the best way to mesh them with the current manuscript.

8. The authors say:
"In particular, we group the shots in our test set based on their predicted probability, and for each set, we estimate the fraction of them that were actually made."

I am confused. What grouping? This should be explained clearly from the outset.

We would like to apologize for any confusion. In this part of the manuscript our objective is to evaluate the shot make probability model. In order to do that we rely on what is termed as calibration curve. Ideally, in order to evaluate a probabilistic prediction p(s) for a shot s, we would need to take that shot 100 times and count how many times the shot was made. If our probabilistic prediction was “accurate”, then we would have expected to see p(s) makes. However, taking the exact same shot - during live ball action - 100 times is not possible. This is why we take all the data points/shots in our validation/hold out set and put them into bins based on the predicted shot make probability from our model. For example, all shots for which we predicted a 55%-60% chance of being made will be placed within the same bin. Then using the data from that bin, we can estimate two quantities; (a) the (average) predicted shot make probability for all the shots in the bin, p_1, and (b) the observed predicted shot make probability

[1] Also this is player-dependent as well, with some players not exhibiting this difference, or exhibiting a difference in the opposite direction. Nylon Calculus has an interactive table where the data from different players are presented: https://fansided.com/2016/02/23/introducing-free-throw-splits/

for the bin p_2 (which is simply the FG% for the shots falling into the bin under examination). In order for the model to be well-calibrated and the probability output accurate, we need to have $p_1 \approx p_2$. We have updated that part of the manuscript to make it more clear.

Other Comments:
1. It is not that the streak selection bias "could lead to underestimation of the hot hand phenomenon", rather that it does lead to the underestimation.

We have changed the phrasing accordingly.

2. The authors say:
"Inset (B) at Figure 1 presents the reliability curve for our shot probability model as obtained through an (out-of-sample) test set."

Which one? Why just one?

Given the setting we included in the first revision of the manuscript in order to expand the number of seasons analyzed, the test set includes all the shots in our dataset. In particular, the 2013-14 season forms the test set for the model trained on the 2014-15 season and vice versa. Figure 1B is presenting the results from the evaluation on these out-of-sample predictions. Possibly the easiest way to describe this is as a two-fold cross validation setting, where the folds are defined by the season. We have updated the revised manuscript to clarify this point.

3. Need to explicitly state how many simulations of the heterogenous Bernoulli process were performed. "...repeatedly simulate" is too vague. Also the statistical testing procedure should be explained clearly.

We have made these updates in the revised manuscript, where in particular we have added a supplementary material section to describe the procedure of the statistical test as detailed as possible.

4. The authors say:
"We use players with at least 1000 shots during the two seasons. Given that there are 82 regular season games in each season, this means that we filter out from our analysis players that took approximately less than 6 shots per game. This threshold was chosen in order to provide, on average, sequences from individual games that can be used to examine the hot-hand hypothesis for values of k > 1. As we will see in our results, we are able to examine up to k = 4."

What is this claim based on? It needs to be backed up by some evidence. Presumably the authors performed a power analysis, and they are summarizing the results here. They need to explain the results of the power analysis in a clearer way. They also suggest elsewhere that they are sufficiently powered. Based on what analysis?

We would like to thank the reviewer for this comment, which allows us to clarify our line of thought behind the choice of this threshold. We did not perform a statistical power analysis. A simple way to explain this threshold choice is that it is a necessary but not sufficient condition to perform tests for values of k up to 4. As we mention in the manuscript a threshold of 1,000 shots over two seasons leads to players with an average of a little less than 6 shots a game. If a player takes less than 6 shots in a game finding “data points” for this player with more than (k=)4 consecutive made shots - in the same game - will be very rare (indeed when we tried to look at k=5, we ended up with an average set of eligible shots for a player less than 10). So while this threshold is not a sufficient condition for having a well powered test, it is necessary for having one at all.

5. One cannot "calculate the probability" of Kemba Walker making a shot. Should fix the language.

We would like to thank the reviewer for this comment. We have gone over the paper again and we hope we have improved/fixed the language.

6. The authors say:
"Furthermore, in the studies by Miller and Sanjurjo on controlled shooting (both actual three-point contests [19] and a shooting field experiment [20]) they also examine whether there is hot hand activated between the different contest rounds. They achieve this by considering sequences of consecutive makes that might span different competition rounds."

This is not quite representative of Miller and Sanjurjo's approach. They first permute on the player level, to facilitate comparison with the previous literature's results, then on the player x session (or round) level, which does two things. First it eliminates vulnerability in the estimates to any systematic variation between sessions (not due to the hot hand), but if any hot hand effect at least partially activates between sessions or rounds it eliminates that too. By contrast, they are not actively trying to "examine whether there is a hot hand activated between the different contest rounds."

We would like to thank the reviewer for this detailed explanation. We have updated the part where we discuss these studies and we believe we have avoided misrepresenting their work in the revised manuscript.

7. The authors say:
"Our results from two seasons of shooting data indicate that overall the league is subject to shooting regression, i.e., players shoot below expectation after consecutive makes, thus, regressing towards their shooting average. However, there are players that exhibit strong statistical evidence for the presence of the hot hand individually."

This writing is not clear. First, the first statment is about the average or representative shot, across shooters. I don't understand the use of "regression towards their shooting average" here. Which shooting average?

We are simply referring here to the general phenomenon of “regression towards the mean”, according to which if a sample point is *extreme*, a future point is likely to be closer to the mean of the process. In our case, if a player scores, which is an “overperformance” according to the shot make probability (which is always < 1), according to the regression to the mean phenomenon his future shots are more likely to be missed, so his overall FG% performance moves closer to his personal/true average FG% (which is certainly less than the 100% observed in his last k consecutive makes). So essentially, what our results say is that if we pool together all the players, the league exhibits this reversion to the mean -- a shot taken after consecutive made shots is made at a percentage less than the expected per the shot make model. However, specific individuals show strong signals for the opposite phenomenon - which is the hot hand. We have rephrased this part in the revised manuscript to hopefully make our intended content more clear.

8. The authors say:
"An important context that we have to add in the hot hand analysis in actual game situations is that the presence of hot hand does not necessarily have to do with what fans might have in mind when they talk about a \player getting hot". It can be simply the ability of specific players to hunt and exploit good matchups for them within a game, leading to a streak of successful shots" and later "exploiting missmatches..."

I don't understand what the authors are trying to express here. What this example brings to mind to me is that the short list of controls the authors use makes their results vulnerable to possible confounds, such as the type of strategic variables they are alluding to, that is not controlled for in their analysis. I´m not sure why they assume this lack of control would inflate rather than deflate estimated hot hand effect sizes. They should clarify this discussion. Bocskocsky et al, Lantis and Nesson, Miller and Sanjurjo, and perhaps Rao, have pointed out this limitation when working with field goal data.

We would like to thank the reviewer for this comment. We have updated our discussion on this in the revised manuscript but here is a more compact explanation. As mentioned in our answer to the previous comment, we simply refer to the observation that as a whole, when we pool together all the league players, a shot following (consecutive) make(s) is missed more often than expected from the shot probability model, *balancing* the players’ recent *overperformance.* What we are referring to here as a possible underlying mechanism for the players that exhibit the hot hand, stems from the purely strategic nature of the game. For example, if a team has decided to counter the opponent’s pick-and-roll with a particular coverage, this can create specific mismatches that the offense can exploit. Let’s assume (a true example from last season’s NBA playoffs) that Doncic is running the pick-and-roll and the opposing team decides

to “switch”. This leads to Zubac matching up with Doncic. Even though Zubac is overall a good defender (so his model coefficient(s) will be indicative of a good defender overall) he provides a great advantage to Doncic with his ball handling and passing abilities. This type of mismatches can be exploited to a short extent - until the opposing team adjusts its strategy - and it is hard to include as controls (as we do not have all this information). Note, that while we control in the model for Zubac’s ability, this is just his overall/average ability, which can be different in specific situations.

With regards to the controls in our model, certainly adding more controls can improve the performance of the model, but according to Lantis and Nesson (page 27 and Table 5 of the NBER working paper) adding controls beyond the shooting player’s behavior does not provide any significant improvements[2]. Unfortunately they do not provide the detailed coefficients and standard errors for each of the independent variables considered. Similarly, Bocskocsky et al. do not provide the detailed results of their regression in order to see the relative importance of each additional covariate. In conclusion, while adding more covariates could provide predictive improvements, existing literature supports that these benefits will be minimal, so we choose to stay with the less complex model. Of course, there are variables that can significantly improve the performance of a shot make probability model (as we discuss in the manuscript) but these require information for the trajectory of the ball, a piece of information that we do not have access to. Finally, to the best of our understanding none of the existing work (Lantis and Nesson, Bocskocsky et al. etc.) have controlled/considered strategic features similar to the situation we described in the previous paragraph (which is to be expected since it is hard to identify and codify them).

9. The point that the authors attribute to Bocskocsky et al (2014) about players taking incrementally difficult shots on a streak of makes was made previously by Rao (2009). As mentioned above, the authors should cite Rao's important work.

We would like to thank the reviewer for this comment, which we agree with. However, we did not make this point in the latest manuscript. We might have made this comment in the original version of the manuscript, which was removed after the first revision. However, according to the earlier comment from the reviewer we have added a discussion on the work by Rao (2009) in the revised manuscript.

10. I do not understand how controlled shooting settings, or Stephen Curry shooting at practice, or the NBA three point shootout, are not evidence of shooting performance in the "real world," as the authors claim. I believe what they mean to say is that it is in the real world but not in games. Then, game basketball settings are still basketball settings, so I think the authors should be careful not to overstate the applicability of their results, or unnecessarily make implicit critiques of controlled shooting studies. After all, controlled experiments play a pretty important role in science in general, and the

[2] Based on our own experience as well with shot make probability models, the main signal is captured by the two distances, player effects (and mainly shooter’s -- not defenders) and shot type.

trade-off between studying game and controlled shooting data is pretty obvious for people who understand a bit of statistics.

Here is another example:
"While there is literature that has examined streaks in real environments such as career trajectories and professional success [11]"

We certainly did not want to implicitly criticize controlled shooting studies. What the reviewer mentions is exactly what we were trying to convey; “in-game”. Controlled shooting studies are real-world experiments. We have made this clarification/distinction in the revised manuscript and used the phrase “in-game” instead of “real-world”.

11. Should say a "large number of permutations", as in 10,000 or 25,000, rather than "a number of permutations" which is a bit too vague. Should explain clearly, somewhere, exactly how many.

We have updated this part of the manuscript -- and other locations -- to specifically mention the number of permutations/simulations performed.
